# Heteromerization of Dopamine D2 and Oxytocin Receptor in Adult Striatal Astrocytes

**DOI:** 10.3390/ijms24054677

**Published:** 2023-02-28

**Authors:** Sarah Amato, Monica Averna, Diego Guidolin, Cristina Ceccoli, Elena Gatta, Simona Candiani, Marco Pedrazzi, Michela Capraro, Guido Maura, Luigi F. Agnati, Chiara Cervetto, Manuela Marcoli

**Affiliations:** 1 Department of Pharmacy, Section of Pharmacology and Toxicology, University of Genova, Viale Cembrano 4, 16148 Genova, Italy; 2 Department of Experimental Medicine, Section of Biochemistry, University of Genova, Viale Benedetto XV 1, 16132 Genova, Italy; 3 Department of Neuroscience, University of Padova, Via Gabelli 63, 35122 Padova, Italy; 4 DIFILAB, Department of Physics, University of Genova, Via Dodecaneso 33, 16146 Genova, Italy; 5 Department of Earth, Environment and Life Sciences, University of Genova, Viale Benedetto XV 5, 16132 Genova, Italy; 6 Department of Biomedical, Metabolic Sciences and Neuroscience, University of Modena and Reggio Emilia, Via Campi 287, 41125 Modena, Italy; 7 Interuniversity Center for the Promotion of the 3Rs Principles in Teaching and Research (Centro 3R), 56122 Pisa, Italy; 8 Center of Excellence for Biomedical Research, University of Genova, Viale Benedetto XV 9, 16132 Genova, Italy

**Keywords:** heterodimers, astrocyte processes, striatum, neuroglia, glutamate, rat, molecular modelling

## Abstract

The ability of oxytocin (OT) to interact with the dopaminergic system through facilitatory D2-OT receptor (OTR) receptor-receptor interaction in the limbic system is increasingly considered to play roles in social or emotional behavior, and suggested to serve as a potential therapeutic target. Although roles of astrocytes in the modulatory effects of OT and dopamine in the central nervous system are well recognized, the possibility of D2-OTR receptor-receptor interaction in astrocytes has been neglected. In purified astrocyte processes from adult rat striatum, we assessed OTR and dopamine D2 receptor expression by confocal analysis. The effects of activation of these receptors were evaluated in the processes through a neurochemical study of glutamate release evoked by 4-aminopyridine; D2-OTR heteromerization was assessed by co-immunoprecipitation and proximity ligation assay (PLA). The structure of the possible D2-OTR heterodimer was estimated by a bioinformatic approach. We found that both D2 and OTR were expressed on the same astrocyte processes and controlled the release of glutamate, showing a facilitatory receptor-receptor interaction in the D2-OTR heteromers. Biochemical and biophysical evidence confirmed D2-OTR heterodimers on striatal astrocytes. The residues in the transmembrane domains four and five of both receptors are predicted to be mainly involved in the heteromerization. In conclusion, roles for astrocytic D2-OTR in the control of glutamatergic synapse functioning through modulation of astrocytic glutamate release should be taken into consideration when considering interactions between oxytocinergic and dopaminergic systems in striatum.

## 1. Introduction

Astrocytes are recognized to play crucial roles in brain connectomics in physiological and in pathological conditions. In particular, astrocytic processes are involved in bidirectional neuron-astrocyte communication, responding to neuronal activity, regulating synapse efficiency, synapse coverage and transmitter diffusion, as well as releasing synaptic and long-distance signals [1,2,3]. At the tripartite synapses, the processes regulate the synaptic glutamate concentration through a balance of the activity of the excitatory amino acid transporters EAAT and of the xc^-^ exchange [4,5], together with the release of the gliotransmitter glutamate, also in a vesicular Ca^2+^-dependent manner [6]. 

In the striatum, astrocytic glutamate release and uptake are suggested to influence the efficacy of glutamatergic synaptic transmission, also on a long-term scale, and the diversity of astrocytes is supposed to contribute to the diversity of presynaptic modifications involving striatal glutamatergic dysfunction in pathological conditions [7]. Indeed, in the dorsal striatum, distinct subpopulations of astrocytes—in response to cortical stimulation [8]—release glutamate that activates N-methyl-D-aspartate (NMDA) receptors on specific medium spiny neurons and metabotropic glutamate receptors at distal synapses [9]. In ventral striatum astrocyte-neuron signaling is well established; astrocytes respond to neurotransmitters with Ca^2+^ increases and release of gliotransmitters—including glutamate and ATP/adenosine—then modulating neuronal activity and synaptic transmission [10]. Accordingly, increasing evidence indicates that striatal astrocytes may be involved in pathological conditions. Indeed, evidence has accumulated supporting a direct involvement of striatal astrocytes in diverse aspects of behavior, including cognition, emotion, and motor and sensory processing [11] in drug abuse disorder [12], or in neurodegenerative disorders such as the Parkinson’s disease (PD, [13,14,15]; see also [16]). 

Oxytocin (OT) is a peptide hormone functioning as a “stress-coping molecule” [17] with a major role in mammalian behavior and health, the effects of which on social-emotional behavior, emotional networks dysfunction, or drug abuse disorder have been hypothesized via actions at the striatum [18,19,20]. Interaction between OT and dopamine in brain areas including the striatum has been recognized to play roles in socio-affiliative behaviors and in the associated disorders [21,22,23]. While OT was previously believed to act exclusively on neurons, increasing evidence indicates the existence and functional relevance of OT receptors (OTRs) on astrocytes (see [24,25,26,27]).

Interestingly, heteromers formed by the dopamine D2 receptor and OTR in striatal neurons were suggested to be a molecular mechanism for OT impact on social and emotional behavior [18,28]. We reported that D2 receptors for dopamine, as well as OTR for OT, could regulate the release of glutamate from the striatal astrocyte processes [27,29,30]. Here we investigate whether a D2-OTR receptor-receptor interaction (RRI) can also be established at the level of the plasma membrane of striatal astrocytes. In particular, here we investigate if OTR and D2 receptors can be expressed by the same astrocytes and at the level of the astrocyte processes, and if the receptors can interact through an RRI. The receptor function was assessed by measuring the effects of receptor activation on the release of glutamate from astrocyte processes obtained from adult rat striatum. The ability of native striatal astrocytic D2 and OTR to heteromerize was assessed by co-immunoprecipitation and proximity ligation assay (PLA). To predict the possible structure of the D2-OTR heterodimer and the residues mainly involved in the heteromerization, a molecular modeling approach was followed.

## 2. Results

### 2.1. Activation of D2 or OT Receptor Inhibits the 4-AP-Evoked Glutamate Release

**Figure 1:**
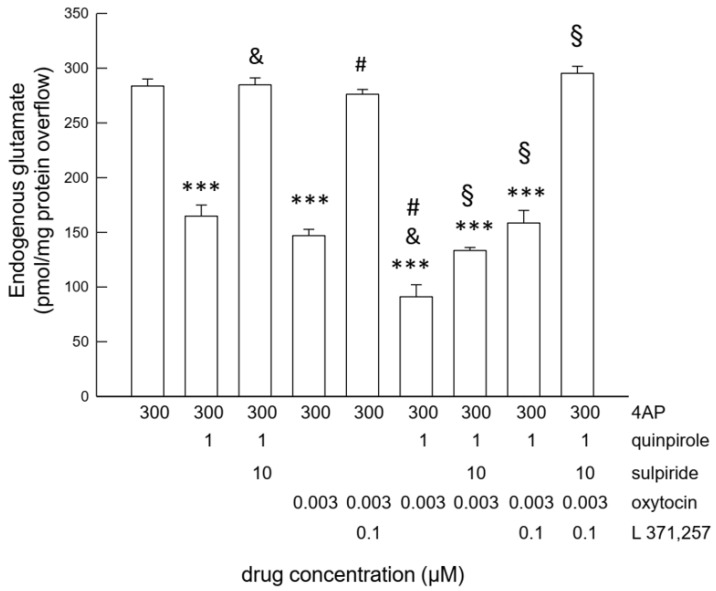
Endogenous glutamate release in response to 4-AP-induced depolarization in striatal gliosomes. Inhibition by OT and D2 receptor activation. Inhibitory effect of OT 3 nM and of quinpirole 1 μM on the 4-AP-evoked endogenous glutamate release; antagonism by L 371,257 0.1 μM and sulpiride 10 μM, respectively; effect of OT 3 nM plus quinpirole 1 μM and antagonism by D2 and OTR antagonists. Bars represent the overflow of the endogenous glutamate release, expressed as pmol/mg of protein, in the presence of the drugs at the concentrations indicated in the figure. 4-AP was added (6 min) during superfusion; OT and/or quinpirole were added together with 4-AP. The antagonists were added 8 min before the agonists. Other experimental details are in Materials and Methods. Data are means ± SEM (bars) of *n* = 3–9 independent experiments. *** *p* < 0.0001 compared with the effect of 4-AP; & *p* < 0.0001 vs. 4-AP + quinpirole; # *p* < 0.005 vs. 4-AP + OT; § *p* < 0.001 vs. 4-AP + quinpirole + OT according to one way ANOVA plus multiple comparison analysis. 4-AP, 4-aminopyridine; L 371,257, 1-[4-[(1-Acetyl-4-piperidinyl)-oxy]-2methoxybenzoyl]-4-(2-oxo-2H-3,1-benzoxazin-1(4H)-yl)-piperidine; OT, oxytocin; OTR oxytocin receptor.

The basal endogenous glutamate outflow in the first two fractions collected from astrocyte processes in superfusion amounted to 88.11 ± 2.65 pmol/mg protein min (*n* = 16). 4-aminopyridine (4-AP; 300 µM) increased the glutamate efflux (4-AP 300 µM evoked overflow: 271.95 ± 6.56 pmol/mg protein; *n* = 16). Activation of the D2 receptor by quinpirole (1 µM) or of the OTR by OT (3 nM) inhibited the 4-AP-evoked efflux of endogenous glutamate in a way sensitive to the D2 receptor antagonist sulpiride (10 μM) and to the OTR antagonist L 371,257 (0.1 µM), respectively (Figure 1), consistent with previous findings [27,29]. OT (3 nM) or quinpirole (1 μM) had no effect on the basal glutamate efflux. Notably, concomitant administration of both the D2 and the OTR agonists together exerted a profound inhibition of the 4-AP-evoked endogenous glutamate efflux, greater than administration of either receptor agonist alone (Figure 1). Sulpiride (10 μM) or L 371,257 (0.1 µM) only partly inhibited the response to concomitant activation of both the D2 and OTR receptors, while the two antagonists together abolished the response to combined activation of both the receptors (Figure 1).

Collectively, the findings indicate that activation of OT receptors or of D2 receptors was able to inhibit the glutamate release in striatal astrocyte processes, and the effect of receptor activation was not mutually exclusive.

### 2.2. Both D2 and OT Receptors Are Co-Localized on Striatal Astrocytic Processes

**Figure 2:**
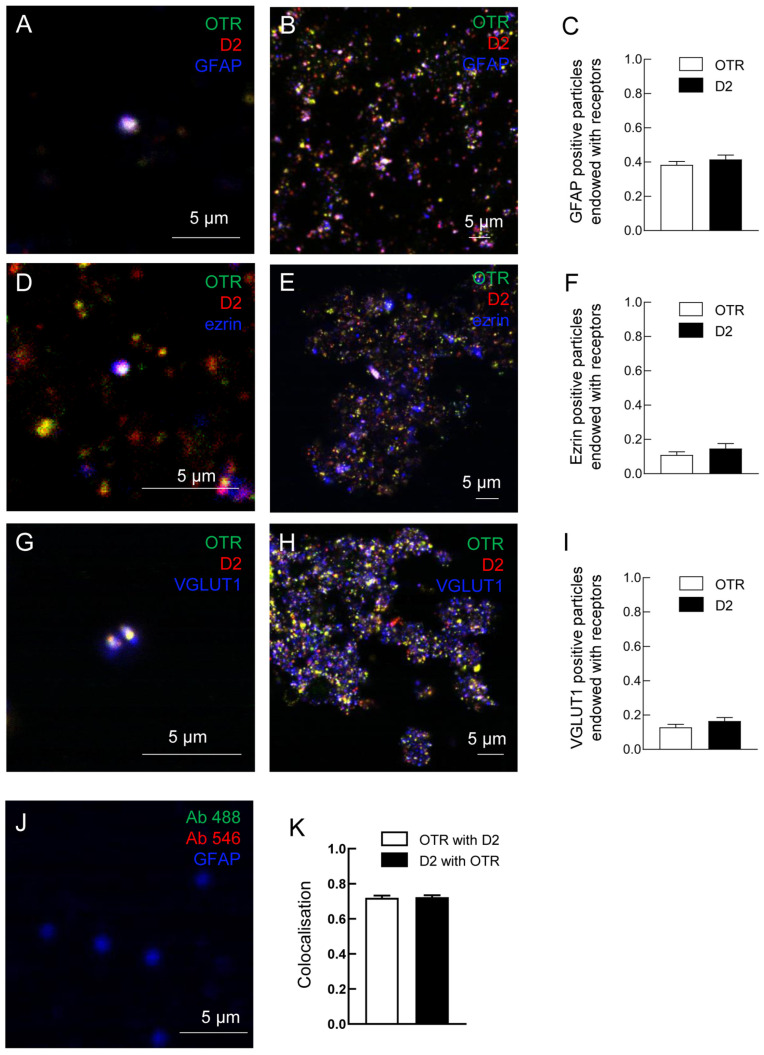
Striatal gliosomes co-express D2 and OT receptors. (**A**–**J**). Representative confocal images showing the co-localization of D2 and OT receptors on gliosomes. Triple immunofluorescence labeling in striatal gliosomes with antibody against the oxytocin OT receptor (**A**,**B**,**D**,**E**,**G**,**H**, green), the dopamine D2 receptor (**A**,**B**,**D**,**E**,**G**,**H**, red) and markers for the glial processes, GFAP (**A**,**B**,**J** blue), or for the PAPs, ezrin (**D***,***E**, blue), or for the vesicular glutamate transporter type 1, VGLUT-1 (**G***,***H**, blue). Representative merged images are shown. Control images were acquired on samples in which the primary antibodies for OTR and D2 receptors were omitted (**J**). Note single gliosomes expressing OTR and D2 (**A**,**D**,**G**). The bars (**C**,**F**,**I**) indicate the percentage (± SEM of 12 fields from three different preparations) of GFAP (**C**) ezrin (**F**), and VGLUT1 (**I**)-positive processes expressing OTR or D2. The bars (**K**) indicate the percentage (±SEM of 40 fields from ten different preparations) of co-localization between the receptors. GFAP, glial fibrillary acidic protein; OT, oxytocin; OTR oxytocin receptor; VGLUT-1, vesicular glutamate transporter type 1.

Astrocytic processes proved to be positive for the astrocytic fibrillary acidic protein (GFAP) markers, for the preferential fine perisynaptic astrocyte processes (PAPs) marker ezrin, and for the vesicular glutamate transporter type 1 (VGLUT1). GFAP-positive, ezrin-positive, or VGLUT1-positive processes were labeled with anti-OTR and with anti-D2 antibodies (Figure 2), indicating that the processes express both D2 and OT receptors (Figure 2). Single astrocyte processes expressing both D2 and OTR are shown at higher magnification in Figure 2A,D,G.

The findings indicate that the same astrocyte processes are endowed with both OT and D2 receptors, therefore possibly allowing a D2-OTR receptor-receptor interaction in striatal astrocytes.

### 2.3. Functional Interaction between OT and D2 Receptors Expressed on Striatal Astrocytic Process

**Figure 3:**
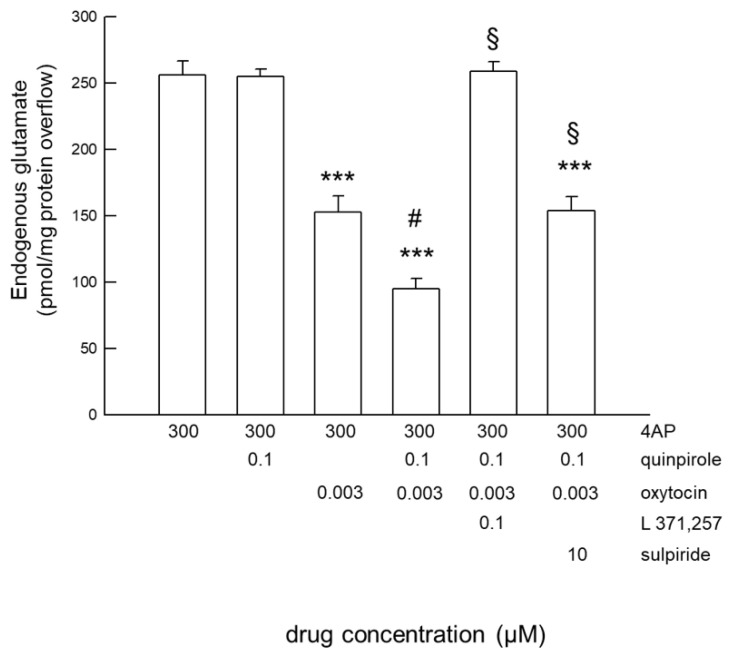
Functional D2-OT receptor-receptor interaction in striatal gliosomes. Effect of quinpirole 0.1 μM on the 4-AP-evoked endogenous glutamate release in the absence and in the presence of OT 3 nM; unmasking a response to quinpirole 0.1 μM in the presence of OT 3 nM; antagonism by sulpiride and L 371,257. Bars represent the endogenous glutamate overflow in the presence of the drugs at the concentrations indicated. 4-AP was added (6 min) during superfusion; OT and/or quinpirole was added together with 4-AP. The antagonists were added 8 min before the agonists. Other experimental details are in Materials and Methods. Data are means ± SEM (bars) of *n* = 3–7 independent experiments. *** *p* < 0.0001 compared with the effect of 4-AP; # *p* < 0.01 vs. 4-AP + OT; § *p* < 0.01 vs. 4-AP + quinpirole + OT according to one way ANOVA plus multiple comparison analysis. 4-AP, 4-aminopyridine; OT, oxytocin.

The effects of combined activation of OTR and D2 receptors were further evaluated. In the presence of OT (3 nM), quinpirole at a concentration (0.1 μM) per se ineffective on glutamate release was able to inhibit the release (Figure 3). The D2 receptor antagonist sulpiride prevented this effect of quinpirole (0.1 μM) in the presence of OT (3 nM), confirming that it was accounted for by D2 receptor activation. The OTR antagonist L-371,257 (0.1 µM) abolished the effect of quinpirole (0.1 μM) in the presence of OT (3 nM), as well as the effect of OT, indicating that activation of the D2 receptor by quinpirole at subthreshold concentration was accounted for by the facilitatory effect of OTR activation on D2 receptor activation.

The findings indicate a functional interaction between OTR and D2 receptors in rat striatal astrocyte processes, showing that activation of OTR had a facilitatory effect on the response of D2 receptors, making them activated by subthreshold D2 agonist concentrations (facilitatory RRI of D2-OTR on D2 receptor-mediated response).

### 2.4. OT and D2 Receptors Expressed on Striatal Astrocytic Processes Physically Interact

The capability of D2 and OT receptors on striatal astrocytes to physically interact was investigated by co-immunoprecipitation assay on purified striatal astrocyte processes. By co-immunoprecipitation we found that the D2 and the OT receptors expressed on the striatal astrocytic processes physically interact.

**Figure 4:**
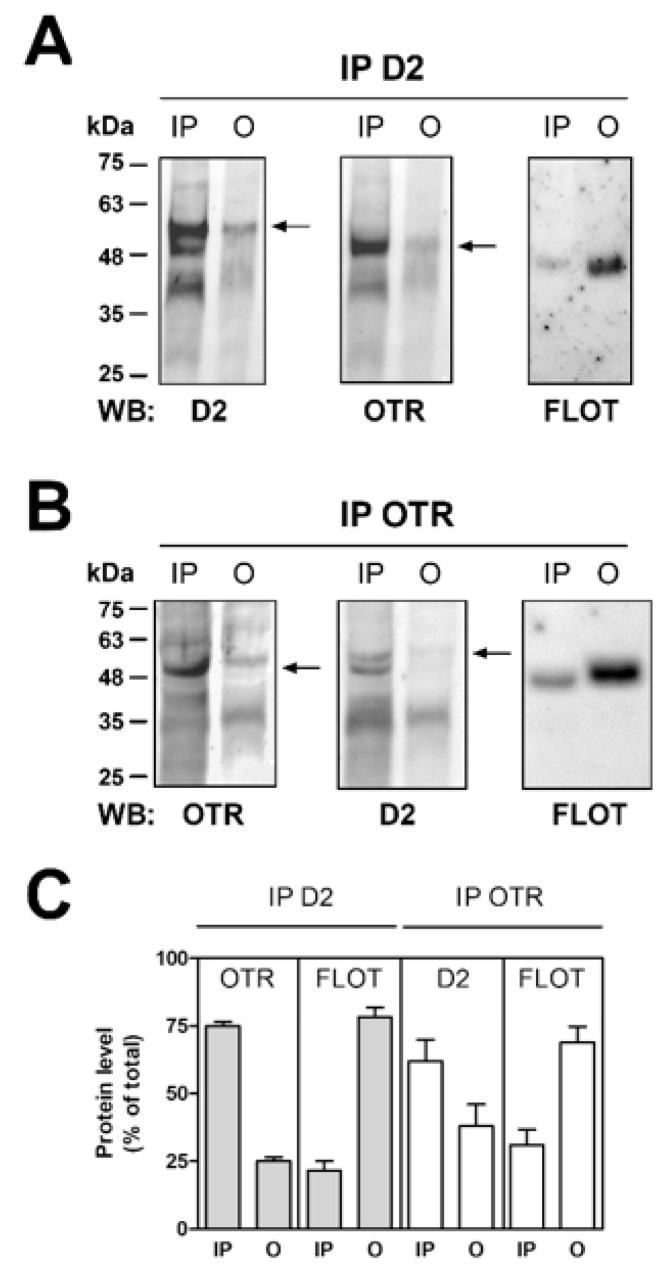
Co-immunoprecipitation of D2 and OT receptors in striatal gliosomes. (**A***)* Aliquots (300 µg) of Triton X-100-soluble proteins prepared from striatal fresh isolated gliosomes were immunoprecipitated with 1 µg of anti-D2 receptor (D2) antibody (see Materials and Methods). The immunoprecipitated (IP) and not immunoprecipitated (O, output) materials were analyzed by immunoblotting using the anti-D2 antibody. IP and O were also analyzed using anti-OTR and the anti-flotillin-1 (FLOT) antibodies. In the figure a representative blot (of five) is shown. The black arrows indicate the expected weights of the antigens. (**B**) Aliquots (300 µg) of Triton X-100-soluble proteins obtained from striatal fresh isolated gliosomes were immunoprecipitated with 1 µg of anti-OTR antibody (see Materials and Methods). IP and O were analyzed by immunoblotting using the anti-OTR antibody. IP and O were also analyzed using anti-D2 and the anti-FLOT antibodies. In the figure a representative blot (of three) is shown. The black arrows indicate the expected weights of the antigens. (**C**). Co-immunoprecipitated OTR, D2 receptor, and FLOT were quantified, and the data were reported as percentage of the total amount of the relevant protein (% of total). Values of co-immunoprecipitated OTR and FLOT with anti-D2 antibody are means ± SEM (*n* = 5), values of co-immunoprecipitated D2 receptor and FLOT with anti-OTR antibody are means ± SEM (*n* = 3). FLOT, flotillin; OTR, oxytocin receptor.

The findings indicate that the OT receptor expressed on the striatal astrocytic process is associated with the D2 receptor. Moreover, a fraction of flotillin-1, a marker of the membrane lipid rafts [31], co-immunoprecipitated with both the OT and the D2 receptors (Figure 4A,B), suggesting that the receptor complexes were enriched in lipid rafts.

### 2.5. OT and D2 Receptors Expressed on Striatal Astrocytes Can Form Heteromers

**Figure 5:**
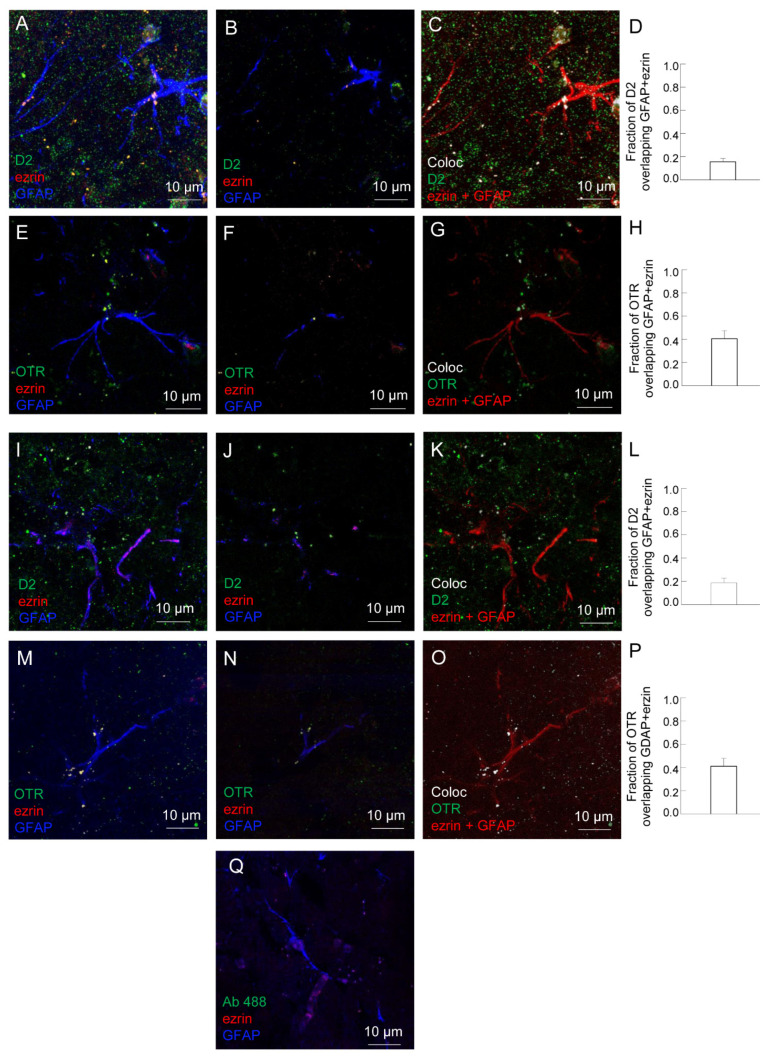
Striatal astrocytes express OT receptors and D2 receptors. Representative confocal images showing the presence of the OTR and D2 receptors in rat ventral (**A**–**H**) or dorsal (**I**–**P**) striatum. Triple immunofluorescence analysis was conducted using primary antibodies: anti-D2 receptor (**A**–**C**,**I**–**K**), anti-OTR (**E**–**G**,**M**–**O**), anti-GFAP and anti-ezrin (**A**–**Q**) in rat hemibrain slices. (**A**,**E**,**I**,**M**) The merge of the maximum intensity projections of a representative field (65 × 65 µm; z 8–12 µm) is shown; GFAP (blue), ezrin (red), D2 (green, **A**–**C**,**I**–**K**) or OTR (green, **E**–**G**,**M**–**O**). (**B,D**,**F**,**H**) Merged confocal images of a single z stack of the image shown in (**B**,**F**,**J**,**N**). (**C**,**G**,**K**,**O**) Co-localized maps of the same images shown in (**A**,**E**,**I**,**M**), and in which ezrin and GFAP were merged and are red, while the receptors are green. The white dots are the co-localized pixels. The bars (**D**,**H**,**L**,**P**) indicate the fractions (±SEM of *n* = 3 fields from three different preparations) of D2 or OTR expressed on GFAP, ezrin, or GFAP + ezrin positive astrocytes. (**Q**) A representative control image acquired on slices in which the primary antibody for OTR or D2 was omitted. GFAP, glial fibrillary acidic protein; OTR, oxytocin receptor.

**Figure 6:**
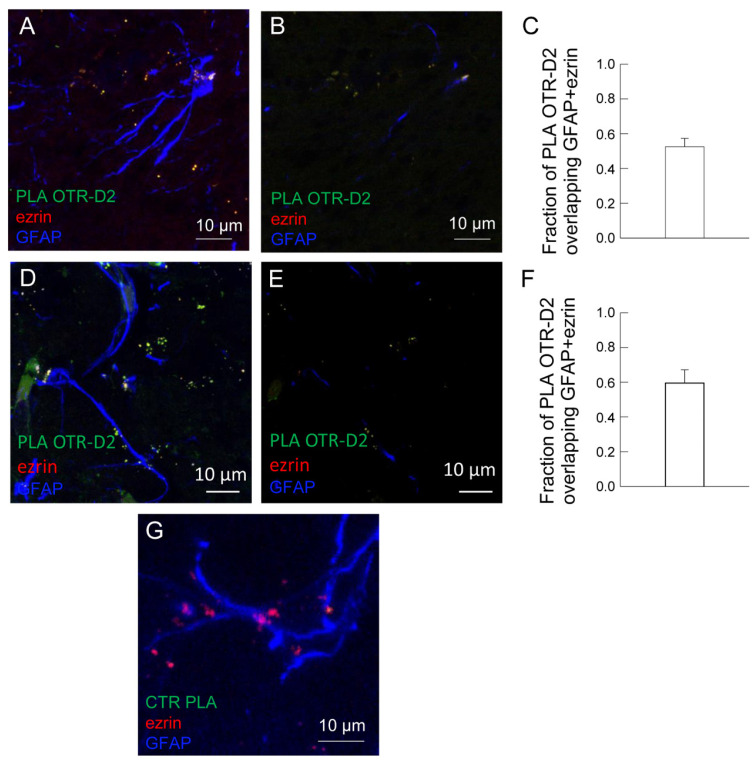
Striatal astrocytes express D2-OTR heterodimers. Representative confocal images showing the presence of the D2-OTR heterodimers in rat ventral (**A**,**B**) or dorsal (**D**–**E**) striatum. In situ Proximity Ligation Assay (PLA) for the D2-OTR heteroreceptor complexes was carried out in rat hemibrain slices using anti-D2R and anti-OTR. The primary antibodies were conjugated with PLA oligonucleotides (PLUS or MINUS) before the PLA experiments, accordingly to the manufacturer’s instructions. For details see Materials and Methods. (**A**,**D**) The image is the merge of the maximum intensity projections of a representative field (66 × 66 and 72 × 72 µm; z 10–14 µm); PLA for D2-OTR heterodimers (green), ezrin (red), GFAP (blue). (**B**,**E**) A single z stack of the image is shown. The bars (**C**,**F**) indicate the fractions (± SEM of *n* = 5–8 fields from three different preparations) of PLA OTR-D2 expressed on GFAP, ezrin, or GFAP + ezrin positive astrocytes. (**G**) In the control, a complete lack of stain for PLA D2-OTR heteroreceptor complexes was obtained. The control condition was conducted using only a primary antibody conjugated with PLA oligonucleotides (PLUS or MINUS). The image is the merges of the maximum intensity projections of a representative field (30 × 30 µm; z 14 µm). Scale bars are shown in the images. GFAP, glial fibrillary acidic protein; OTR, oxytocin receptor; PLA, proximity ligation assay.

In ventral and dorsal striatum, astrocytes were identified by the astrocyte markers GFAP and the PAPs marker ezrin (see Figure 5A–Q). The presence of D2 and OTR on striatal astrocytes in both ventral and dorsal striatum was assessed by immunofluorescence (Figure 5A–Q). The co-localization analysis showed that 15.6 ± 2.9 % and 18.8 ± 3.9 % of D2 receptors appeared localized on astrocytic structures (positive for GFAP, ezrin, or GFAP plus ezrin) in ventral and dorsal striatum, respectively; the same analysis indicated that 40.4 ± 6.9 % and 41 ± 6.8 % of OTR appeared localized on astrocytes in ventral and dorsal striatum, respectively (see Figure 5D,H,L,P). Their ability to heteromerize was tested by proximity ligation assay (PLA; Figure 6A–E). The in situ PLA assay showed green spots for D2-OTR heterodimer complexes in GFAP-positive astrocytes and in ezrin-positive fine PAPs in both the ventral and dorsal striatum, as shown in a maximum intensity projection in single z stack (Figure 6). The heterodimer co-localization on astrocytic structures in ventral and dorsal striatum was also quantified (Figure 6C,F). As negative controls, we performed the experiment with only one of the two primary antibodies, and PLA signal was not detected (Figure 6G). 

Altogether these data support the existence of D2-OTR heteromers in the astrocytes of rat ventral and dorsal striatum.

### 2.6. Estimated Model of the D2-OTR Heterodimer

**Figure 7:**
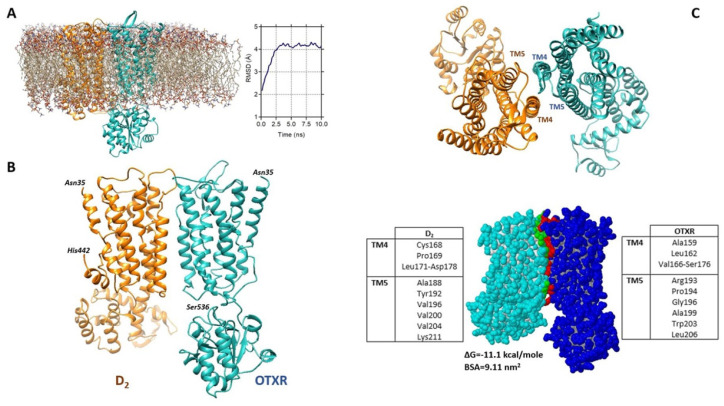
Estimated model of the D2-OTR heterodimer. (**A**) The heterodimeric structure of D_2_ and OTR, as obtained by docking, is shown in the environment used for Molecular Dynamics, including a 1-palmitoyl-2-oleoylphosphatidylcholine (POPC) lipid bilayer, ions (sodium and chloride, 0.15 M), and water molecules (not shown). The RMSD trajectory during the production phase is also shown. (**B**) Heterodimeric complex between D_2_ and OTR as predicted by the computational procedure used. (**C**) In the upper panel the extracellular side of the estimated heteromeric structure is shown to indicate the TM4 and TM5 domains. The interface between D_2_ and OTR is illustrated in the bottom panel together with the residues mainly involved in its formation. The ΔG for complex formation and the interface area (buried surface area, BSA), as predicted by PDBePISA, are also reported. OTR, oxytocin receptor; RMSD, root mean square deviation; TM, transmembrane domains.

As shown in Figure 7A, the overall root mean square deviation (RMSD) of the Cα atoms of the dimer increased at the beginning of the unrestrained phase of the simulation and then stabilized at about 4.2 Å, indicating that the structure reached a stable conformation at 300 °K. The obtained structure is shown in Figure 7B.

As reported in Figure 7C, the residues predicted to be mainly involved in the heteromerization interface are located in the transmembrane domains four and five (TM4 and TM5) of both D_2_ and OTR.

## 3. Discussion

A key finding of the study is that OT, through a facilitatory allosteric D2-OTR RRI in striatal astrocyte processes, permits activation of the D2 receptor by subthreshold dopamine agonist concentration; this may be of great potential relevance in conditions of striatal dopamine deficit. To our knowledge, the study provides the first evidence for heteromerization of native OT and D2 receptors in astrocytes. In detail, the following novelties are reported: both OT and D2 receptors are expressed on the same astrocytes and, in particular, on the same astrocyte processes in striatum, including GFAP-positive branches/branchlets and ezrin-positive PAPs; the astrocytic OT and D2 receptors functionally interact and control glutamate release from the processes; astrocytic OT and D2 receptors can form receptor heteromers in both the ventral and dorsal striatum. The evidence was obtained in slices from adult rat striatum and in astrocyte processes acutely prepared from astrocytes matured in the astrocyte-neuron network, therefore reflecting the features of the processes in striatal neuron-astrocyte networks in the adult brain. Striatal gliosomes were previously shown to represent a preparation of astrocytic processes with negligible neuronal contamination (see also [29,30]). Functional evidence also indicated that the gliosomes prepared from adult rat striatum were not contaminated by glutamate-releasing nerve terminals: A2A receptor activation which increased the glutamate release from the nerve terminals obtained in parallel was completely ineffective on either basal or stimulated glutamate release from gliosomes [29]. The striatal gliosomes were positive for the astrocytic marker GFAP or for ezrin, a selective marker restricted to the perisynaptic processes [32], required for their structural plasticity [2,33]. Notably, perisynaptic processes appear to be crucially involved in the control of glutamate uptake and release at synapses (see [34,35] and references therein); the gliosome positivity for ezrin is therefore consistent with their origin from perisynaptic processes and their ability to release glutamate. Indeed, gliosomes maintain the machinery for glutamate exocytotic release and expressed VGLUT1, which is indicative for the presence of glutamatergic vesicles; both D2 and OTR were co-expressed on processes endowed with the vesicles (co-expressing VGLUT1). The presence of vesicles in striatal astrocyte processes had been previously confirmed by ultrastructural analysis: smooth vesicles with a 30 nm diameter and with the morphological characteristics of the astrocytic vesicles capable of fusion ([30]; see also [36]) displayed scattered distribution in the gliosome cytoplasm. Consistent with our ex vivo findings, synaptic-like vesicles carrying VGLUT1 have been found in rat striatum both within larger astrocyte processes and in the perisynaptic astrocyte processes [37]. In agreement with vesicular glutamate release, we found that the 4-AP-evoked glutamate release from the processes was Ca^2+^-dependent [29]; the finding is consistent with the ability of in situ striatal astrocytes to release glutamate upon an increase of their intracytoplasmic Ca^2+^ levels [9]. Taken together, the findings suggest that processes of mature rat striatal astrocytes are capable of vesicular exocytotic release of glutamate that can be evoked by 4-AP depolarization.

Notably, 4-AP is a selective blocker of Kv channels (see [38]). As a matter of fact, Kir4.1, a weakly inwardly rectifying K^+^ channel, represents a major astrocyte K^+^ channel which appears primarily responsible for astrocyte hyperpolarization [39]; putative functions of the channel include K^+^ homeostasis, maintenance of the astrocyte membrane potential, cell volume regulation, and regulation of glutamate fluxes [40]. Additional subtypes of inward-rectifying K^+^ channels have been characterized in astrocytes beyond Kir4.1. In particular, several types of voltage-dependent K^+^ (K_V_) channels have been identified [41]. Kv channels were found to play a role in the regulation of astrocytic membrane potential and Ca^2+^ influx, suggesting that they can modulate the astrocyte excitability and gliotransmitter release [42]. Interestingly, these voltage-dependent K^+^ channels exhibit distinct subcellular localization; the Kv3.4 subtype was expressed primarily in the astrocyte processes whereas the Kv4.3 was found localized to the somata [43,44]. Notably, the Kv3 family channels are sensitive to 4-AP submillimolar concentrations (see [41,45]); the 4-AP glutamate-releasing effect might therefore be related to blockade of Kv in the astrocyte processes. Accordingly, the 4-AP evoked release from the processes was found to be dependent on Ca^2+^ influx [29].

### 3.1. D2 and OT Receptors Are Expressed on the Same Striatal Astrocyte Processes

Confocal imaging showed that striatal astrocytes expressed both D2 and OTR; both the receptors were expressed on the same astrocytes and astrocyte processes, opening the possibility of direct RRIs at the striatal astrocyte plasma membrane. Activation of either the D2 or the OTR receptor could inhibit the evoked glutamate release from striatal processes, suggesting that both oxytocin and dopamine through OTR and D2 receptors, respectively, possess the capability to regulate glutamatergic transmission in striatal neuron-astrocyte networks, their effects being not mutually exclusive.

### 3.2. OT and D2 Receptors on Striatal Astrocyte Processes Functionally Interact

OTR and D2 receptors were found to interact functionally in an RRI, through which OT was able to facilitate D2 receptor activation. Indeed, when OT was concurrently bound to astrocytic OTRs, the astrocytic D2 receptors could be activated (mediating a functional response) by an otherwise ineffective low concentration of the D2 receptor agonist quinpirole. The finding is in accordance with the increased affinity of dopamine to D2 receptors when OT was concurrently bound to OTR in striatal neurons [18,28]. The ability of OT to make effective dopamine levels otherwise too low to activate the astrocytic D2 receptor might be relevant for a better understanding of the control of glutamatergic transmission in striatum.

The facilitatory allosteric D2-OTR RRI in striatal neurons [18] was regarded as a molecular mechanism for the OT ability to induce changes in social and emotional behavior [18,28]. Notably, involvement of dopamine/oxytocin interaction on social behavior like motivation and partner preference formation in animals has been proposed [46]; the interaction between DA and OT in the nucleus accumbens is essential for pair-bonding behavior in both male and female prairie voles [47]. Abnormal OT-DA interactions may contribute to behavioral disorders such as autism, sexual dysfunction, addiction and depression, and risk for post-traumatic stress disorders [21,48]. The OT-mediated social affiliative behaviors seem linked to regulation of the dopaminergic reward system [49,50]. So, it is perhaps not surprising that the dopaminergic system is involved in the mechanism(s) underlying the effect of OT in modulating addictive-related behaviors [51]. Interestingly, activation of D2 receptor reduces drug-seeking behavior, and OT may reduce drug-seeking behavior via interactions with D2 receptors in the nucleus accumbens [52]. By considering the relevance of astrocytes in social and emotional behavior [24,53], we hypothesize that facilitatory allosteric D2-OTR RRI in astrocytes may contribute to the OT impact on social and emotional behavior.

### 3.3. Astrocytic OTR and D2 Receptors Can Form Receptor Heteromers

OTR was suggested to dimerize with the D2 receptors in striatal neurons, and OTR activation increased the affinity of the D2 receptor for dopamine, though the dimerization sites were unknown [18,28]. Our findings indicate that native OT and D2 receptors expressed on the striatal astrocyte processes can undergo RRI based on receptor heteromerization. Co-immunoprecipitation of the receptors indicates that the RRI was based on a physical interaction. While most OTR immunoprecipitated together with the D2 receptor, only a fraction of the D2 receptor co-immunoprecipitated together with the OTR, suggesting that at striatal astrocyte processes the D2 may remain as monomers, or possibly interact also with other receptor types. Indeed, the D2 receptor [54] as well as OTR [27] was found to co-immunoprecipitate together with A2A, indicating that these receptors may build higher order receptor complexes. Co-immunoprecipitation of OT and D2 receptors with the membrane lipid rafts marker flotillin-1 [31] suggests enrichment of the receptor complexes in lipid rafts, which may provide the ordered membrane microenvironment for horizontal molecular networks of G-protein-coupled receptors (GPCRs) complexes [55,56] in the astrocytic process membrane. PLA analysis confirmed that OTR and D2 receptors form heteromers on GFAP-positive striatal astrocytes and on the ezrin-positive fine processes.

The estimated model of the D2-OTR heterodimer indicated that the structure could reach a stable conformation, and predicted that the residues mainly involved in the heteromerization interface are located in the transmembrane domains four and five (TM4 and TM5) of both D2 and OTR. We previously reported that in the striatal astrocyte processes, native D2 and OTR receptors can heteromerize with the A2A receptor [27,29,30,54]. It remains to be clarified if the D2 and OT receptors may also form higher level heteromers, and by which mechanisms, in striatal astrocytes.

### 3.4. Potential Relevance of Striatal Astrocytic D2-OTR Heteromers

It appears important here to recall that the facilitatory D2-OTR RRI in striatal astrocytes resulted in the ability of OT to make effective on glutamate release dopaminergic agonist subthreshold concentrations otherwise too low to activate the astrocytic D2 receptor, of potential relevance in PD where dopamine is low at striatal level. Anti-neuroinflammatory and neuroprotective effects of OT might be as well related to a facilitatory interaction with dopamine at astrocytic D2 receptors. The facilitatory D2-OTR RRI would be relevant not only for a better understanding of the control of glutamatergic transmission in striatum, but especially for new potential therapeutic approaches to pathological conditions such as PD, in which morphological changes of striatal astrocytes, neuroinflammation, and striatal glutamatergic transmission dysregulation are reported [13,14,57,58]; see also [30]. Interestingly, allosteric modulators of receptors, permitting greater receptor selectivity and reduced side effects, are recognized of relevant therapeutic potential in central nervous system (CNS) disorders [59,60]: targeting the facilitatory allosteric D2-OTR RRI may provide greater receptor and temporal selectivity, offering a way to increase the selectivity of pharmacological treatments and decrease the adverse side effects.

Changes of striatal astrocytes with PAPs expansion and increased coverage of glutamatergic synapses are reported in PD ([13,57]; see also [30]). Notably, OT was reported to affect the PAPs motility by retracting PAPs and regulating coverage of the synapse [61,62,63]; see [64], and in supraoptic nucleus the astrocyte morphology, GFAP’s plasticity, and PAPs retraction dynamically reflected OT neuronal activity [65,66]. Ezrin is well known to be involved in the structural changes required for PAPs motility and regulation of synapse coverage [32,33]; the presence of OTR and D2-OTR heteromers on ezrin-positive striatal PAPs suggests that OT can be involved in the control of astrocytic coverage of striatal synapses. By this way OT, by regulating the ability of neurotransmitters to diffuse to extrasynaptic long-distance targets [67,68,69,70], may be crucial to the balance and integration of wiring and volume transmission [71,72,73,74] in the striatal integrative functions and might restore a dysregulation in PD. Therefore, OT might impact on striatal glutamate transmission dysregulation by balancing glutamate wiring and volume transmission, as well as by controlling glutamate release from the processes and facilitating the D2-mediated control.

It appears also worth mentioning that OT can be released mostly non-synaptically, and seems to act mainly through volume transmission by diffusing at distance from the sites of release [75,76]. Interestingly, dopamine can also be released non-synaptically and act through volume transmission in striatum ([77,78] and references therein; see also [73,79]). Therefore, the reports suggesting the OT ability to control astrocytic coverage of the synapses [63,65,66] could be relevant to understand its wide modulatory effects and their implication for the complexity of signal integration in the brain, and for the relevance to OT and dopamine interaction, in this case specifically in striatum, in physiological and in pathological conditions.

Chronic neuroinflammation and astrocytic and microglial activation are considered hallmarks of PD [80] and are proposed to play roles in PD pathophysiology [81,82,83], although the exact mechanisms involved in the relationship between neuroinflammation and PD remains to be elucidated [84]. Astrocytes are increasingly recognized as being able to influence dopaminergic neuron degeneration, exerting both neuroprotective and neurotoxic actions, and are proposed as a promising strategy target to control the progression of PD (see [85,86]; see also [87,88,89]). Notably, glial D2 receptors seem to play important roles in the modulation of neuroinflammation and in the maintenance of immune homeostasis, and ablation of astrocytic D2 receptor caused reduction of anti-inflammation protein alphaB-crystallin in the CNS ([90], see also [91,92]). Reduced signaling of astrocytic D2 receptors could increase the striatal dopaminergic neuron’s vulnerability to MPTP (1-methyl-4-phenyl-1,2,3,6-tetrahydropyridine; [90]), and down-regulation of D2 receptors in the aging brain has been suggested to compromise the immune homeostasis, contributing to PD pathogenesis [80]. In fact, D2 receptors were also reported to play neuroprotective roles in the brain (cortex, striatum) against injury-induced neuroinflammation, neurodegeneration, and synaptic dysfunction [93,94]. Interestingly, OT was reported to possess anti-neuroinflammatory and neuroprotective effects involving glial cells: OT could attenuate glial activation induced by lipopolysaccharide and reduce expression of proinflammatory mediators and cytokines [95]. Our findings suggest that OT might therefore contribute to rescue an impaired anti-neuroinflammatory effect also resulting from a defective activation of astrocytic D2 receptors.

Altered neuron-astrocytic interactions at the striatal glutamatergic synapses, with increased levels of glutamate (see [58] and references therein) have been implicated in the pathophysiology of PD, and glutamatergic overactivity is proposed as a critical mechanism underlying striatal alterations in early and advanced symptomatic stages of PD (reviewed in [96]). In fact, the astrocyte dysfunction, also linked to altered control of glutamatergic transmission, was suggested to have an initiating role in the pathophysiology of PD [14]. Notably, we here confirm that both astrocytic D2 receptors (see [29,30]) and OT receptors (see [27]) may play roles in the control of glutamatergic transmission in striatum, and propose that OT might rescue the impaired control of striatal glutamatergic transmission played by a defective activation of astrocytic D2 receptors.

Indeed, the facilitatory D2-OTR RRI resulting in a facilitation of the activation of the astrocytic striatal D2 receptor might be related to a potential usefulness of OT in PD. Consistently, a reduction of the number of OT-immunoreactive neurons in the hypothalamus of PD patients [97], and the ability of OT to influence locomotor activity at the level of the substantia nigra [98], possibly suggest that a decrease of OT levels in the brain areas receiving oxytocinergic projections may play a role in the motor disabilities in PD patients [98]. Accordingly, OT was reported to possess neuroprotective effects on rotenone-induced PD in rats [99,100] or in MPTP-treated mice [101]. In fact, OT could allow the astrocytic D2 receptor’s activation by low extracellular levels of dopamine and could rescue the reduced activation of the D2 receptors (by a defective dopaminergic transmission in PD). OT therefore could help astrocytic D2 receptors to control the glutamate level at the striatal synapse by rescuing glutamatergic transmission dysregulation, and to play neuroprotective roles also by releasing anti-neuroinflammatory factors.

PD is recently proposed as a complex neuropsychiatric disorder, as in many cases neuropsychiatric symptoms including impulse control disorders, with associated risk factors such as doses/duration of dopamine agonists treatment, novelty-seeking or impulsivity traits, and history of substance use disorder, which are recognized to be of similar relevance as the motor symptoms; the search for safe efficacious treatment for the neuropsychiatric disorders is a research priority in PD [102]. Notably, OT effects on social-emotional behavior and potential effectiveness in dysfunction of the emotional networks have been hypothesized via actions at striatum [18,19,20]. Therefore, the potential of OT receptor to ameliorate D2 response on striatal astrocytes might represent a new field worth exploring in the search for a multidrug approach to PD, and to some of the neuropsychiatric symptoms in PD, possibly also allowing a reduction of doses/duration of dopamine agonists treatment.

In fact, OT by a facilitatory effect on D2 receptor activation at astrocyte level, might help to maintain dopaminergic neuron function in early PD, to delay the onset of PD symptoms related to defective dopamine receptor activation, and to make effective low doses of PD medications. The potential clinical usefulness of OT as adjunctive drug therapy in PD patients would be therefore based also on the possibility to reduce the dopaminergic therapy side effects, including the impulsivity disorders mainly in patients with addictive behaviors. As a matter of fact, evidence in animal PD models is provided that OT administration may possess neuroprotective effects on dopaminergic neurons related to anti-inflammatory, antioxidant, and anti-apoptotic activities [103], that dopamine levels increased when OT was administrated [104], that the OT levels were decreased in the models, that OT supplementation rescued locomotor disabilities and anxiety-like behaviors [101], and that after intranasal administration OT concentrates in brain regions including the striatum [105]; nevertheless, studies investigating OT in PD clinical settings are still needed [106]. In fact, critical examinations appear fundamental for enabling the utilization of OT mechanisms as a curative tool in psychiatric disorders, in particular when considering that the understanding of how OT impacts social behavior is still insufficient, life experience can change the OT systems function, and the OT effects appear highly context-dependent [107]. In addition, assessing the effects of intranasal OT in patients would need proper dose-response studies, including control subjects for peripheral effects [108]. Notably, a small pilot study in PD patients suggested that intranasal OT could improve motor impulsivity, which might be reflected by a clinical improvement of overall impulsivity, particularly in PD patients with addictive behaviors [109]. Such preliminary results of course warrant validation. 

## 4. Materials and Methods

### 4.1. Animals

For the described experiments, adult male Sprague–Dawley rats weighing approximately 200–250 g were used. Animals were housed at the animal care facility of the Department of Pharmacy (DIFAR), University of Genova, Italy. The rats were kept in control condition at 22 ± 1 °C, 50% relative humidity, and with a 12 h light/dark cycle, with light from 7 AM to 7 PM. The rats had free access to standard diet and water ad libitum. The animal care and the experimental procedures were carried out according to the European Communities Parliament and Council Directive of 22 September 2010 (2010/63/EU) and with the Italian D.L. n. 26/2014. The experimental procedures were approved by the Italian Ministry of Health (protocol number 30/11/2016-OPBA of November 2016), in accordance with Decreto Ministeriale 116/1992. All efforts were made to minimize animal suffering and to reduce the number of animals used.

### 4.2. Preparation of Purified Astrocytic Processes

After decapitation, striatum was dissected and rapidly placed in ice-cold medium. As previously reported [29,110], the purified astrocyte processes (gliosomes) were prepared according to Nakamura’s protocol [111]. Striatum was homogenized in 0.32 M sucrose and 10 mM Tris/HCl (pH 7.4) using a grinder and the homogenate was centrifugated (5 min at 1000× *g*; 4 °C) to remove nuclei and debris. A second centrifugation (5 min at 33,500× *g*; 4 °C) on a discontinuous Percoll gradient (2, 6, 10 and 20% (*v*/*v*) in Tris-buffered sucrose allowed to collect the gliosomes at the layer between 2% and 6% (*v*/*v*) Percoll. The gliosmes were then washed by centrifugation in the final HEPES standard medium for the release experiments, immunofluorescence (IF) analysis, or the co-immunoprecipitation experiments. The standard medium was buffered at pH 7.4 and had the following composition (mM): NaCl 128, KCl 2.4, MgSO_4_ 1.2, KH_2_PO_4_ 1.2, CaCl_2_ 1.0, and HEPES 10 with glucose 10. Gliosomes obtained this way are purified astrocyte processes with negligible neuronal contamination, containing gliotransmitter-loaded vesicles and capable of gliotransmitter secretion [29,36,112]. Superfusion of a gliosomal monolayer, avoiding receptor biophase and any indirect affect that substances released from the particles might have, therefore allowing substances added to the perfusion medium to act upon the so called “nude receptors” makes it an optimized model to characterize gliotransmitter-released modulating receptors [29,113].

### 4.3. Endogenous Glutamate Release

As previously reported [27,114], we studied the release of endogenous glutamate from the gliosomes using the up-bottom superfusion technique. The gliosomes were stratified as a monolayer at the bottom of superfusion chambers and superfused at 0.5 mL/min with medium at 37 °C. The standard medium could be supplemented with pharmacological tools (agonists and antagonists) and so this technique allows the pharmacological characterization of release regulating receptors present on the gliosomes, and the interaction that could be intercurred among receptors [27,29,30]. During the superfusion, 3 min superfusate samples (from B1 to B5) were collected for each chamber starting at t = 33 min. At t = 38 the gliosomes were exposed to the depolarizing stimulus (300 µM 4-AP; 6 min). The D2 agonist and/or OT were added together with 4-AP; when we evaluated the antagonist effects, the antagonists were added 8 min before the agonists. For control, in each experiment at least one chamber was superfused only with standard medium or with medium appropriately modified (e.g., added with antagonists).

For the pharmacological characterization of D2 receptor mediated responses we used quinpirole as an agonist and sulpiride as the antagonist, at the concentrations indicated in the figures; for OTR-mediated responses we used OT as agonist and L371,257 as antagonist. The drug concentrations were chosen as follow: to depolarize the astrocyte processes, the submaximal 4-AP concentration (300 mM; see [45]) was chosen; the same concentration was previously used to evoke vesicular exocytotic transmitter release from the processes [29,30]. The concentrations of quinpirole were chosen as they proved to inhibit the 4-AP evoked glutamate release through a selective D2 receptor activation, as indicated by effectiveness of the D2 receptor antagonist sulpiride (see also [29,30]). The selected OT concentration (3 nM) was effective in inhibiting the 4-AP evoked glutamate release through OT receptor activation, lower (1 nM) or higher (10 nM) concentrations being ineffective (see [27]). Notably, the very same concentration could facilitate the D2-mediated response in neuronal D2-OT heteromers as well, while lower or higher concentrations were reported to be ineffective (see [18]).

The amount of glutamate released in the collected samples was measured by HPLC, as previously described [27,115]. The HPLC-based amino acid reverse-phase chromatography analysis involved an automatic precolumn derivatization (Waters Alliance; Milford, MA, USA), the use of an internal standard, the separation on a C18 column, and the fluorimetric detection. An *O*-phthalaldehyde solution was used as derivatization reagent, while homoserine was used as the internal standard. The detection limit of this analytical technique was 100 fmol/mL. For each experiment the protein determinations were carried out using Bradford’s method [116] and the glutamate amount released in the collected samples (B1-B5) was expressed as pmol/mg protein. For each superfusion chamber, the mean of glutamate amount released in the two basal superfusate samples (B1 and B2) was taken as the 100% control value. The amount of the endogenous glutamate released in the other fractions (B3–B5) was then evaluated as the percent variation with respect to the corresponding control value. The effect of the used drugs in the release of glutamate was measured by subtracting the percent variations of glutamate efflux in control conditions (standard medium or medium added with antagonist) from the percent variations in drug-presence conditions.

### 4.4. Immunofluorescent Labelling in Gliosomes

For immunofluorescence confocal analysis, gliosomes (1 μg/μL) were resuspended in standard HEPES medium and the experiments were carried out as previously described [27,29,112]. For each sample, 15–20 µg of gliosomes were fixed in a solution of paraformaldehyde 2% in phosphate buffer saline (PBS), permeabilized for 5 min with 0.05% Triton X-100 in PBS containing 3% bovine serum albumin (BSA) and then incubated O.N. at 4 °C with the primary antibodies diluted in PBS supplemented with 3% BSA. To avoid the cross-reaction among the primary antibodies a sequel protocol was applied. The primary antibodies used were goat anti-GFAP (1:500; Santa Cruz Biotechnoloy Inc., Dallas, TX, USA), rabbit anti-D2 receptor (1:200; Alomone Labs, Jerusalem, Israel), rabbit anti-OTR (1:200; Alomone Labs), mouse anti-ezrin (1:100; Sigma-Aldrich, Milan, Italy), and guinea pig anti-vesicular glutamate transporter 1 (VGLUT1; 1:500; Merck Millipore Corporation, Milan, Italy). After the incubation with a primary antibody and three times washing with PBS, the gliosomes were incubated (60 min at room temperature) with a secondary antibody Alexa Fluor 488, 546, or 633 conjugated (1:1000; Life Technologies Corporation, Carlsbad, CA, USA) and diluted in PBS containing 3% BSA. The labelled gliosomes were placed on slides and prepared for imaging using ProLong Gold mounting medium. Clear nail polish was be used to seal the edges of coverslips to slides. The slides were stored at 4 °C until confocal analysis. In parallel, negative control slides were prepared avoiding the presence of a primary antibody during the experimental procedure but using the relative secondary conjugated-antibody; the result was a complete lack of the relative stain (see also, [27,54]).

### 4.5. Immunoprecipitation and Immunoblot

Gliosomes obtained from striata of four animals were suspended in 50 mM sodium borate buffer, pH 7.5 with 1 mM EDTA, and Protease Inhibitor Cocktail (lysis buffer) at 1 mg/mL and lysed by three cycles of freezing and thawing followed by sonication. Protein quantification of lysate was performed using the Bradford method [116]. To perform the immunoprecipitation, gliosome lysate was centrifuged at 100,000× *g* for 30 min at 4 °C. The pellet was washed once and then solubilized in 100 µL of 50 mM sodium borate, 0.1 mM EDTA, pH 7.5 (immunoprecipitation buffer) + 1% Triton-X at 37 °C for 1 h. Then 400 µL of immunoprecipitation buffer was added to the lysate to dilute the Triton-X to 0.2% (total of the membranes) and then centrifuged at 18,000× *g* for 15 min at 4 °C. The supernatant was precleared with protein G-sepharose, and then incubated in the presence of 1 µg of anti-D2 receptor antibody or anti-OTR antibody at 4 °C, overnight. Protein G-sepharose was then added to the samples and incubated for an additional 1 h at room temperature (RT). The immunocomplexes were centrifuged at 400× *g* and aliquots of supernatant were submitted to SDS-PAGE. The immunocomplexes were washed three times with immunoprecipitation buffer + 0.1% Triton-X, heated in SDS-PAGE loading buffer for 5 min and submitted to 10% SDS-PAGE followed by electroblotting onto a nitrocellulose membrane and saturated with phosphate-buffered saline, pH 7.5, containing 5% skim milk powder. The blots were probed with specific antibodies and the immunoreactive material was detected with a Bio-Rad Chemi Doc XRS apparatus.

### 4.6. Striatal Slices Preparation

Immediately after decapitation, the whole brain was removed, and treated to prepare cryostat sections as previously described [27,117]. The right or left half of the rat brain was embedded in paraformaldehyde 4% in PBS for at least 24 h, at 4 °C, in sucrose 30% in PBS (24 h at 4 °C), in Killik (Bio-Optica, Milan, Italy), an optical cutting temperature compound, and finally frozen in liquid nitrogen. Coronal sections 10–15 µm thick were prepared using a cryostat (Leica CM1900UV, Wetzlar, Germany) and were collected on poly(L)-lysine pre-coated slides. Until further processing the obtained slides were stored at −20 °C.

### 4.7. Proximity Ligation Assay (PLA) and Immunofluorescent Confocal Microscopy on Slices

To assay the physical interaction between OTR and D2 receptors on rat hemi brain slices, in situ Proximity Ligation Assay (PLA) was carried out essentially as previously described [54,118]. Firstly, the rabbit anti-D2 receptor (Alomone Labs) and the rabbit anti-OTR (Alomone Labs) antibodies were conjugated with the PLA PLUS and PLA MINUS oligonucleotides, respectively, using the Duolink in situ Probemaker kit (PLUS DUO92009, MINUS DUO92010, Sigma-Aldrich), following the manufacturer’s instructions. The antibodies were added to Conjugation Buffer, transferred into glass vials containing lyophilized oligonucleotides PLUS or MINUS, and then incubated overnight at room temperature. Subsequently, the conjugation reaction was stopped by adding Stop Reagent. Storage Solution was used to stabilize the conjugated antibodies. PLA was performed using the Duolink Detection Kit (DUO92014, DUO92002, DUO92004 Sigma-Aldrich). Briefly, as indicated in the kit instructions, 10–15 µm slices were washed three times (5 min at room temperature) in PBS, permeabilized with 0.2% Triton X-100 in PBS containing 3% BSA, and then blocked with Blocking Solution. The conjugated anti-D2 PLUS and anti-OTR MINUS antibodies were diluted in Probe Diluent solution 1X and then added on the slices. The incubation with the antibodies was conducted in a humid chamber O.N. at 4 °C. Thereafter, at the manufacture’s instruction planning, the hybridization, ligation, and amplification steps were performed, enabling the visualization of the D2-OTR heteromers by green fluorescence. To co-localize the heteromers on astrocytes after the amplification step, the slices were incubated, washed three time (5 min), and incubated in a humid chamber overnight at 4 °C with goat anti-GFAP (1:500, Santa Cruz Biotechnology Inc., Dallas, TX, USA) and mouse anti-ezrin (1:100; Sigma-Aldrich) diluted in Antibody Diluent solution. The specie-specific secondary antibodies were Alexa Fluor 633-conjugated donkey anti-goat and 546 donkey anti-mouse (incubated for 1 h at RT). The slices were then washed in Wash Buffer B 1X, Wash Buffer B 0.01X, and prepared for imaging using Mounting Medium (DUO82940). Clear nail polish was used to seal the edges of coverslips to slides. The slides were stored at 4 °C until confocal analysis. In parallel, negative control slices were prepared, avoiding the conjugation of the primary antibody with the oligonucleotides, resulting in a complete lack of PLA stain. Moreover, in some slices the specificity of the double immunolabeling was tested and the primary antibodies were replaced with PBS (see also [27,54]).

### 4.8. Confocal Microscopy on Gliosomes and Slices

The labelled gliosomes and slices were analyzed by confocal microscopy using a Leica STELLARIS 8 Falcon τSTED (Leica Microsystems, Mannheim, Germany) inverted confocal/STED microscope. Excitation was provided by a white light laser, selecting the combination of chosen fluorochromes to avoid crosstalk. Detection was performed by three Power HyD detectors. The fluorescence image (1024 × 1024 × 16 bit) acquisition was performed using an HC PL APO CS oil immersion objective 100× (1.40 NA). The pinhole was set to 1 Airy size. Line scanning speed range was 400 Hz. Leica “LAS X application Suite” software package 4.4.0.24861 was used for acquisition, storage, visualization, and 3D analysis.

### 4.9. Co-Localization Analysis

On gliosomes, to co-localize OTR or D2 receptors on GFAP, ezrin, VGLUT1, or each other, we used JACoP plug-in [119] in ImageJ Fiji software 2.9 (Wayne Rasband, National Institutes of Health, Bethesda, MD, USA). This plug-in calculates general commonly used parameters. For a couple of images, if the same pixel and their respective intensities were strictly higher than the threshold of the two channels, for example green (OTR) and red (D2), labels were considered as co-localized. Data were collected from 12 to 40 fields from three to ten independent preparations, and are expressed as mean ± SEM in the bar graphs.

On slices, to co-localize labeling for OTR and D2 receptor, or for PLA signals on astrocytes, we prepared merged images for each z stack of the channels 01 and 02 for ezrin and GFAP labeling, respectively. The merged images were saved as 16-bit and then used for the co-localization analysis between OTR or D2 receptors or PLA, and the glial markers using JACoP plug-in in ImageJ Fiji software, and working on the z stacks as described above for the gliosomes. Data were collected from all the z stacks of three to six independent experiments, and are expressed as mean ± SEM. 

### 4.10. Receptor Structures

Experimentally assessed molecular structures of human adenosine D2 receptor and oxytocin receptor (OTR) were retrieved from the Protein Data Bank (https://www.rcsb.org accessed on 10 September 2021). Both D2 (PDB code: 6CM4 [120]) and OTR (PDB code: 6TPK [121]) were experimentally obtained by X-ray diffraction at a resolution of 2.87 Å and 3.20 Å, respectively.

By using the DockPrep module available in the UCSF Chimera molecular modeling software (Resource for Biocomputing, Visualization, and Informatics, University of California, San Francisco; http://www.rbvi.ucsf.edu/chimera, downloaded 4 June 2021) all extra molecules (such as ligands) were removed, hydrogens were added, and partial charge assigned. The obtained molecular structures were then energy minimized by using the Yasara software (http://www.yasara.org/minimizationserver.htm, accessed 20 January 2022 [122]) and stored for further processing.

### 4.11. Modeling of the D2-OTR Heteroreceptor Complex

Structures of possible heterodimers of D2 and OTR were estimated following a previously reported procedure [123].

Briefly, the GalaxyHeteromer (http://galaxy.seoklab.org/ accessed on 21 January 2022) software was used to perform protein-protein docking [124] and after ranking the solutions by energy score, the best solution exhibiting the correct orientation of the monomers was selected as a possible heterodimeric structure.

To refine the obtained heterodimer using a more realistic model of the biological environment, the structure was inserted into a pre-equilibrated 1-palmitoyl-2-oleoylphosphatidylcholine (POPC) bilayer (see Figure 7A) using CHARMM-GUI Membrane Builder (http://www.charmm-gui.org/?doc=input, accessed on 22 January 2022), a simulation preparation software [125]. Molecular Dynamics simulations were then run (see [123]) using the NAMD package [126] (version 2.12) with the CHARMM36 force field [127], powered by the VMD software [128] for data visualization and management. The system first underwent 1 ns equilibration phase, involving heating from 0 °K to 300 °K, followed by a production (unrestrained) phase of 10 ns. The protein complex configuration at the end of the production phase was taken as predictive of the heterodimer structure and the structural features of the predicted heteromerization interface were explored by the PDBePISA tool [129] freely available at https://www.ebi.ac.uk/pdbe/pisa/ (accessed on 30 January 2022).

### 4.12. Calculations and Statistical Analysis

The numbers of experiments (n) are indicated in the figures. The data are expressed as means ± SEM. The significance of the difference was analyzed by one way ANOVA and multiple comparison analysis, with statistical significance being taken at *p* < 0.05.

### 4.13. Materials

4-Aminopyridine (4-AP), Triton-X 100, quinpirole, sulpiride, and oxytocin (OT) were purchased from Sigma-Aldrich, while L-371,257 was from Tocris. 4-AP and oxytocin were dissolved in distilled water. Sulpiride and L-371,257 were dissolved in DMSO and then diluted 1:1000 in physiological medium. DMSO 0.1% had no effect on endogenous glutamate release from gliosomes. All the salts were from Sigma-Aldrich or from VWR. Protein G-sepharose 4 Fast Flow, nitrocellulose membrane, and ECL Select were obtained from GE Healthcare. The anti-rabbit secondary antibody and the Protease Inhibitor Cocktail were obtained from Cell Signaling Technology.

## 5. Conclusions

Our findings, contributing to a better understanding of roles that astrocytes could play in the regulation of glutamate transmission in striatum, also possibly provide a new target for neuropharmacological treatment of pathological conditions characterized by a dysfunction of the striatal glutamatergic transmission. In fact, although future replication of receptor co-localization in cultured astrocytes might be important, evidence for receptor co-localization on the astrocytes in slices, and on the isolated astrocyte processes, concur to indicate that mature striatal astrocytes indeed express the receptors, and the receptors can undergo heteromerization. By considering that astrocytes and altered astrocyte-neuron intercellular communications at striatal synapses are recognized to play roles in PD ([13,14,15]; see also [16]), our findings help to understand how astrocytes and remodeling of astrocyte processes could contribute to the pathophysiology of PD, how defect of oxytocinergic transmission may contribute to the PD pathophysiology, and how OT might effectively ameliorate the disease. In fact, astrocyte-based treatments for PD are proposed (see [88,89]). The potential of OT receptor to ameliorate D2 response on striatal astrocytes might then be relevant to a new therapeutic approach to PD, and OT-mediated facilitation of D2 receptor-mediated responses would contribute to the control of PD. 

## Data Availability

Data available on request from the corresponding authors.
